# Dissociation in reactive and proactive inhibitory control in Myoclonus dystonia

**DOI:** 10.1038/s41598-020-70926-x

**Published:** 2020-08-18

**Authors:** Cyril Atkinson-Clement, Clement Tarrano, Camille-Albane Porte, Nicolas Wattiez, Cécile Delorme, Eavan M. McGovern, Vanessa Brochard, Stéphane Thobois, Christine Tranchant, David Grabli, Bertrand Degos, Jean-Christophe Corvol, Jean-Michel Pedespan, Pierre Krystkoviak, Jean-Luc Houeto, Adrian Degardin, Luc Defebvre, Romain Valabregue, Charlotte Rosso, Emmanuelle Apartis, Marie Vidailhet, Pierre Pouget, Emmanuel Roze, Yulia Worbe

**Affiliations:** 1 Sorbonne University, 75005 Paris, France; 2 Inserm U1127, CNRS UMR7225, UM75, ICM, 75013 Paris, France; 3 Movement Investigation and Therapeutics Team, Paris, France; 4 Assistance Publique-Hôpitaux de Paris, Centre d’Investigation Clinique Neurosciences, Hôpital Pitié-Salpêtrière, Paris, France; 5 Department of Neurology, Groupe Hospitalier Pitié-Salpêtrière, Paris, France; 6 Department of Neurology, CHU Côte de Nacre, Université Caen Normandie, Caen, France; 7 Inserm, UMRS1158 Neurophysiologie Respiratoire Expérimentale et Clinique, Sorbonne University, Paris, France; 8 Department of Neurology, St Vincent’s University Hospital Dublin, Dublin, Ireland; 9 INSERM/APHP, Centre d’Investigation Clinique 1422, Paris, France; 10 Institut des Sciences Cognitives Marc Jeannerod, CNRS, UMR 5229, University of Lyon, Bron, France; 11 Service de Neurologie C, Hospices Civils de Lyon, Hôpital Neurologique Pierre Wertheimer, Bron, France; 12 Service de Neurologie, Hôpitaux Universitaires de Strasbourg, Institut de Génétique et de Biologie Moléculaire et Cellulaire (IGBMC), INSERM-U964/CNRS-UMR7104/Université de Strasbourg, Fédération de Médecine Translationnelle de Strasbourg (FMTS), Université de Strasbourg, Strasbourg, France; 13 Department of Neurology, Hôpital Avicennes, Assistance Publique-Hôpitaux de Paris, Bobigny, France; 14 Unité de Neuropédiatrie, CHU Pellegrin, Bordeaux, France; 15 Department of Neurology, Amiens University Medical Center, Amiens, France; 16 Service de Neurologie, CIC-INSERM 1402, CHU de Poitiers, Poitiers, France; 17 Department of Neurology, Centre Hospitalier de Tourcoing, Tourcoing, France; 18 INSERM, U1171-Degenerative and Vascular Cognitive Disorders, CHU Lille, Université de Lille, Lille, France; 19 Lille Centre of Excellence for Neurodegenerative Diseases (LiCEND), Lille, France; 20 UMR S 975, CNRS UMR 7225, ICM, Centre de NeuroImagerie de Recherche (CENIR), Sorbonne Université, Paris, France; 21 Assistance Publique-Hôpitaux de Paris, Urgences Cérébro-Vasculaires, Hôpital de la Pitié Salpêtrière, Paris, France; 22 Department of Neurophysiology, Saint-Antoine Hospital, Assistance Publique-Hôpitaux de Paris, Paris, France

**Keywords:** Dystonia, Movement disorders, Neurological disorders, Comorbidities, Neurological manifestations

## Abstract

Myoclonus-dystonia (MD) is a syndrome characterized by myoclonus of subcortical origin and dystonia, frequently associated with psychiatric comorbidities. The motor and psychiatric phenotypes of this syndrome likely result from cortico-striato-thamalo-cerebellar-cortical pathway dysfunction. We hypothesized that reactive and proactive inhibitory control may be altered in these patients. Using the Stop Signal Task, we assessed reactive and proactive inhibitory control in MD patients with (n = 12) and without (n = 21) deep brain stimulation of the globus pallidus interna and compared their performance to matched healthy controls (n = 24). Reactive inhibition was considered as the ability to stop an already initiated action and measured using the stop signal reaction time. Proactive inhibition was assessed through the influence of several consecutive GO or STOP trials on decreased response time or inhibitory process facilitation. The proactive inhibition was solely impaired in unoperated MD patients. Patients with deep brain stimulation showed impairment in reactive inhibition, independent of presence of obsessive–compulsive disorders. This impairment in reactive inhibitory control correlated with intrinsic severity of myoclonus (i.e. pre-operative score). The results point to a dissociation in reactive and proactive inhibitory control in MD patients with and without deep brain stimulation of the globus pallidus interna.

## Introduction

Myoclonus-dystonia (MD) is a syndrome characterized subcortical myoclonus predominating in the upper body, usually associated with dystonia^1^. Mutations in the epsilon sarcoglycan gene (SGCE) are the most common cause^2^. This gene is widely expressed in the cerebellum and the basal ganglia^3,4^ and this mutation likely disrupts the cerebello-thalamo-cortical and cortico-striato-thalamo-cortical pathways^5–8^, presumably leading to inappropriate motor responses in MD^7,9^. In particular, abnormal saccadic adaptation^10^ and altered cerebellar learning^11^, albeit inconsistently^12^, has been observed in these patients. A F^18^-fluorodeoxyglucose PET study found selective metabolic abnormalities in the cerebellum, pons and thalamus in this group of patients, further pointing to dysfunction in these pathways^13^.

Dual pathway alteration is also suggested to underpin psychiatric symptoms frequently observed in MD^14^ such as anxiety disorder, obsessive–compulsive disorders (OCD) or addictions^15,16^. However, little is known about the cognitive abnormalities in MD. The frequent association of MD with OCD and addictions may result from an inhibitory deficit, which was previously identified in these psychiatric disorders^17–19^.

On a behavioural level, the ability to withhold an action is divided into two different, but not independent, processes. Proactive inhibition refers to the ability to inhibit an action in preparation, is generally associated with a dynamic strategy to adapt behaviour and is context-dependent^20,21^. Reactive inhibition refers to the ability to stop an already initiated action and to a lesser degree is related to the context. The Stop Signal Task^22^ provides an opportunity to measure both of these inhibitory processes^23,24^.

On a neuronal level, these two types of inhibitory control are related on distinct, but partially overlapping cortico-striatal networks^25^, including inferior frontal gyrus, precentral gyrus, supplementary motor area and subthalamic nucleus^26,27^ as well as the striatum and globus pallidus interna (GPI)^28^. Interestingly, the GPI is a key structure supporting the inhibitory control of actions and deep brain stimulation of the GPI is a gold standard treatment for motor symptoms in MD^29–31^. However, despite the motor benefit conferred by deep brain stimulation of the GPI in various dystonic syndromes, discrepant results for psychiatric symptoms have been reported^32–37^.

Despite being a potentially promising cognitive marker, inhibitory control, and especially reactive inhibition, has not been extensively studied in MD patients. To date, only one study tested inhibitory control in MD using the Go/NoGo task and demonstrated unaltered inhibitory capacity^38^. The aim of the present study was to evaluate reactive and proactive inhibitory capacity in patients with MD. We also evaluated the relationship between inhibitory capacity and symptom severity and the presence of OCD.

## Results

### Demographic and psychometric results

One unoperated patient and one HC were excluded from the final analysis due to outliers stop signal reaction time value. As shown in Table 1, we found that (1) Unified Myoclonus Rating Scale scores were improved after GPI deep brain stimulation (F_(1;20)_ = 28.114; *p* < 0.001); (2) MD patients with and without deep brain stimulation had a more frequent association with OCD (*p* < 0.001) and had a higher number of impulsive behaviours (Minnesota Impulse Disorders Interview; *p* < 0.001) compared to HC; (3) Unoperated patients only (MD group) had a higher depression score (Beck Depression Inventory; *p* = 0.013) than HC. No significant differences were found between two groups of MD patients (*p* > 0.05).

**Table 1:** Demographics and clinical characteristics of patients and controls.

	HC (n = 24)	MD (n = 21)	MD-DBS (n = 12)		Main effects
			Pre DBS	Post DBS	
Sex (M/F)	14/10	13/8	–	6/6	0.799
Age in years	29.5 ± 10.34	30.05 ± 11.66	–	34 ± 10.51	0.484
Years of education	12.96 ± 1.65	13.14 ± 1.98	–	12.25 ± 1.71	0.379
BFM	–	11.81 ± 7.53	NA	9.13 ± 9.86	0.386
UMRS Total	–	27.43 ± 14.11	73.9 ± 32.89^b^	15.42 ± 17.93^b^	**0.041**
UMRS Action part	–	20.76 ± 11.08	48.5 ± 20.61^b^	13.67 ± 13.49^b^	0.112
UMRS rest part	–	6.67 ± 6.30	25.4 ± 16.61^b^	1.75 ± 5.19^b^	**0.029**
BDI	2.04 ± 2.68	4.89 ± 3.75^a^	NA	4.42 ± 4.79	**0.014**
MIDI	0.12 ± 0.45	1.67 ± 1.56^a^	NA	1.83 ± 1.5^a^	**< 0.001**
OCD (n)	0	6^a^	NA	4^a^	**< 0.001**

All significant *p* (*p* < 0.05) were identified in bold.

*BDI* Beck Depression Inventory, *BFM* Burke–Fahn–Marsden scale, *F* Female, *GPi-DBS* globus pallidus deep brain stimulation, *HC* healthy controls, *M* male, *MD* Myoclonus-dystonia without DBS, *MD-DBS* Myoclonus-dystonia with DBS, *MIDI* Minnesota Impulse Disorders Interview, *NA* not assessed, *OCD* obsessive–compulsive disorders, *UMRS* Unified Myoclonus Rating Scale.

^a^Significantly different from HC.

^b^significantly different pre and post-surgery.

### Reactive inhibition

**Figure 1:**
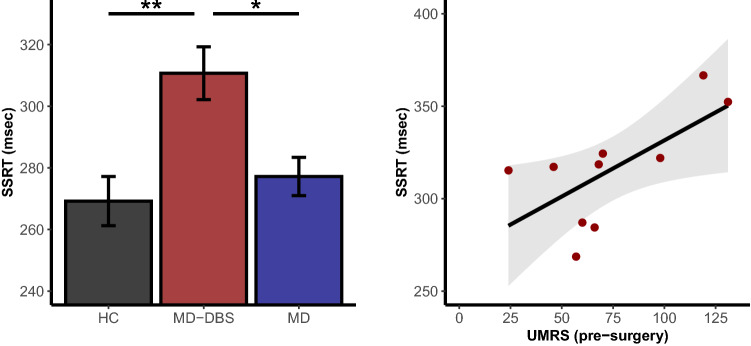
Stop signal reaction time differences between groups (left panel) and correlation with pre-surgery Unified Myoclonus Rating Scale score for the patients with deep brain stimulation (right panel). Two UMRS (pre-surgery) scores were missing. *p < 0.05 after Tukey correction; **p < 0.01 after Tukey correction. *HC* healthy controls, *MD* Myoclonus-dystonia without deep brain stimulation, *MD-DBS* Myoclonus-dystonia with deep brain stimulation, *SSRT* stop signal reaction time, *UMRS* Unified Myoclonus Rating Scale.

Reactive inhibition was measured as the time that participants needed to stop an already initiated action, called the stop signal reaction time (SSRT). We observed a significant main effect of group on stop signal reaction time (F_(2;54)_ = 6.29; *p* = 0.0035), which was driven by MD-DBS compared to HC (*p* = 0.003) and to MD (*p* = 0.022; Fig. 1). The absence of significant difference between HC and MD was confirmed with conventional statistics (*p* = 0.708) and Bayesian approach (Bayesian factor = 0.37 ± 0.02%; anecdotal evidence for null hypothesis). We found no significant effects when comparing MD patients with and without OCD (*p* > 0.05; Bayesian factor = 0.32 ± 0.01%; anecdotal evidence for null hypothesis). As shown in Fig. 1 the stop signal reaction time correlates with the pre-surgery Unified Myoclonus Rating Scale score for the operated patients (r = 0.67; *p* = 0.035) with no outlier detection (*p* = 0.96). This correlation was not significant using Unified Myoclonus Rating Scale actual score with deep brain stimulation ON (r = − 0.39; *p* = 0.21), or when we considered the percentage of improvement due to deep brain stimulation (difference between pre-surgery and actual deep brain stimulation ON score; r = 0.27; *p* = 0.46). No other significant correlation with the stop signal reaction time has been found. All values were summarised in the Table 2.

**Table 2:** Behavioral performances of patients and controls.

	HC	MD	MD-DBS	Main effects
GO accuracy	0.982 ± 0.133	0.988 ± 0.106	0.980 ± 0.139	0.26
STOP accuracy	0.544 ± 0.498	0.548 ± 0.498	0.517 ± 0.499	0.22
GO RT	530.7 ± 144.9	535.3 ± 134.0	509.4 ± 149.1	0.43
Failed STOP RT	439.8 ± 111.1	453.8 ± 92.1	432.9 ± 110.6	0.76
SSRT	269.2 ± 39.1	277.2 ± 28.5^b^	310.7 ± 29.7^ab^	**0.0035**

All significant *p* (*p* < 0.05) were identified in bold.

*HC* healthy controls, *MD* Myoclonus-dystonia without deep brain stimulation, *MD-DBS* Myoclonus-dystonia with deep brain stimulation, *SSRT* STOP signal reaction time.

^a^Significantly different from HC.

^b^Significantly different between patients.

### Proactive inhibitory control

Proactive inhibitory control was assessed as an adaptation of behavioural performances after several consecutive GO or STOP trials under the hypothesis that several consecutive GO trials will facilitate the response process and will decrease reaction time. In contrast, several consecutive STOP trials will facilitate the inhibitory process and increase reaction time^23,24,39,40^.

**Figure 2:**
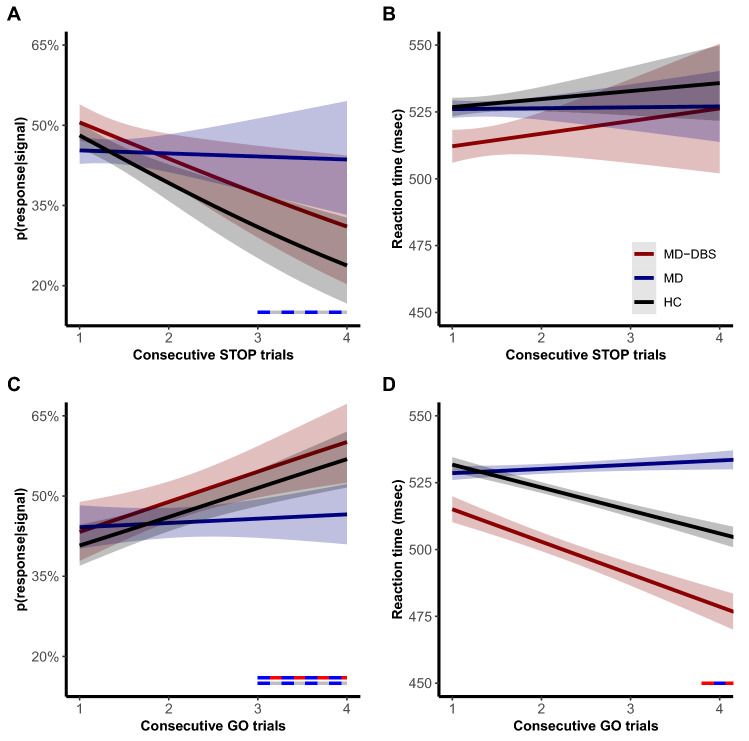
Effects of consecutives STOP (top panels) and GO trials (bottom panels) on p(response|signal) (left panels) and reaction time (right panels) for healthy controls and patients with and without internal globus pallidus deep brain stimulation. The bicolour horizontal lines represent the significant comparisons between two groups according to the number of consecutive GO or STOP trials. *HC* healthy controls, *MD* Myoclonus-dystonia without deep brain stimulation, *MD-DBS* Myoclonus-dystonia with deep brain stimulation, *p*(*response|signal*) probability to failed inhibiting action during a STOP trial, *RT* reaction time.

Reaction time and probability of failure to inhibit action during a STOP trial (p(response|signal)) were not significantly different between the groups (*p* > 0.05). These effects were confirmed by Bayesian approach for reaction time (Bayesian factor = 0.08 ± 0.69%, strong evidence for null hypothesis) and p(response|signal) (Bayesian factor = 0.01 ± 0.88%, very strong evidence for null hypothesis). Nevertheless, as shown in Fig. 2A, we found that p(response|signal) was significantly influenced by the number of consecutives STOP trials (F_(1;5114)_ = 10.56; *p* = 0.0012), with a significant interaction with the group (F_(2;5114)_ = 3.36; *p* = 0.038), but not with OCD (F_(2;5114)_ = 2.46; *p* = 0.089). Precisely, for the p(response|signal), MD patients failed to inhibit response even after three or four consecutives STOP trials in difference with HC (*p* = 0.038) and MD-DBS patients (*p* = 0.044). We found no influence of the number of consecutive STOP trials on the following reaction time and no interaction with the groups (*p* > 0.05; Fig. 2B).

Also, the number of consecutive GO trials before a STOP trial increased the probability of a failure to stop (F_(1;3272)_ = 20.52; *p* < 0.0001), especially in HC and MD-DBS patients (F_(2;3272)_ = 3.69; *p* = 0.027; Fig. 2C). Post-hoc comparison showed that MD patients had a lower chance of stop failure after 3 or 4 consecutive GO trials (*p* < 0.05) in contrast to other two groups of subjects.

There was an effect of number of consecutives GO trials on RT (Fig. 2D) (F_(1;9820)_ = 11.11; *p* = 0.00086), a significant interaction with the groups (F_(2;9820)_ = 7.38; *p* = 0.00062) and no significant interaction with OCD (F_(2;9820)_ = 2.88; *p* = 0.056). There was a significant decrease in RT after the 4th GO trial for HC and MD-DBS, but not in MD patients (*p* < 0.05). Corrected post-hoc comparisons showed a significant difference between MD and MD-DBS after five consecutive GO trials (*p* = 0.044), but not between MD and HC (*p* > 0.05). We did not find significant correlations of these results with clinical data (*p* > 0.05).

## Discussion

We showed two main independent results in patients with SCGE positive MD: (1) proactive inhibition was impaired in MD patients, since they failed to adapt their behavioural performance after several consecutive GO or STOP trials and without alteration of the reactive inhibition; (2) reactive inhibition was impaired in MD-DBS patients as evidenced by longer stop signal reaction times and without alteration of proactive inhibition. These impairments were independent of comorbidity with OCD and correlated to intrinsic severity of the myoclonus (pre-operative score) in MD-DBS group. Noteworthy, we did not find a difference in other task measures such as GO and STOP accuracies or failed STOP reaction time, suggesting that motor symptoms in patients did not interfere with task performance.

As proactive inhibition is defined as the ability to adapt inhibitory performances in a dynamic context, solely unoperated MD patients have an alteration of the proactive inhibition as they failed to change their inhibitory performance and their reaction time following consecutives GO and STOP trials, in contrast to HC and MD-DBS patients. This deficit in proactive inhibitory control was independent of reactive inhibition (no alteration in this patients’ group) and was not correlated with symptom severity or related to the presence of the OCD. This failure to adapt the action was previously reported in the MD patients using various sensori-motor adaptation tasks^10,11^, albeit non-consistently^12^.

Interestingly, the proactive inhibitory capacity of the MD-DBS patients was not significantly different compared to the performance of the HC, suggesting that potentially deep brain stimulation of the GPI could improve this capacity in MD patients. Indeed, the internal globus pallidus was shown to contribute to proactive inhibitory control^41^ and learning aspects based on action-outcome representation^42,43^. On a neuronal level, the pallidal deep brain stimulation effect could also result from partial or complete restoration of activity within the cerebello-thalamo-cortical pathway^44^ via the connectivity of the internal globus pallidus with the thalamus^45^. In contrast to the MD patients, MD-DBS group showed a deficit in the reactive inhibition that could be a result of deep bran stimulation itself and of the severity of the disease as indexed by correlation with pre-operative Unified Myoclonus Rating Scale score.

MD is suggested to be related to abnormalities in the cerebellum, pons and thalamus^13^, which are all involved in motor inhibition as well. For instance, the cerebellum has been linked to stop signal task to STOP errors in HC^46,47^ and to the inhibitory dysfunction assessed with a Go/NoGo task in MD^38^. The cerebellum could influence action inhibitory control through its connections with the subthalamic nucleus via the pontine nucleus and directly to the striatum^48^ as well as via the cerebello-thalamo-pallidal network^46^. This suggests that dysfunction of this network could play a role in both impaired action inhibitory control and myoclonus, which was reinforced by the correlation that we found between reactive inhibition and intrinsic severity of myoclonus in MD-DBS.

Deep brain stimulation, which influences output from the globus pallidus interna^49^, could also induce impairment in reactive inhibition along with improvement of myoclonus as myoclonus severity has been linked to burst activity of the internal globus pallidus in pre-operative studies^8^. Thus, it is plausible that deep brain stimulation of internal globus pallidus might impair reactive inhibitory control, as response inhibition results from a tuned interplay of both hyper-direct and direct pathways^50^. This effect might be also mediated by the striato-nigral pathway, which is influenced by pallidal deep brain stimulation^51^. Previous studies pointed to the activity of the substantia nigra during the stop signal task, specifically during STOP trials^52^.

On the other hand, previous studies showed discrepant results on the effect on action inhibition of deep brain stimulation of the GPI. For example, in patients with Parkinson’s disease and with pallidal stimulation, no effect on stop signal reaction time was reported when referring specifically to reactive inhibition^53^. This would suggest a specific effect of the disease rather than of the deep brain stimulation. To definitively disentangle these two possible explanations, further studies pre- and post-surgery and ON and OFF GPI deep brain stimulation would be warranted. However, a practical issue could be symptom resurgence after switching the stimulation OFF and with subsequent alteration on the task performance or the severity of myoclonus in pre-operative stage that also could highly impact the performance on the task. In addition, evaluation of the current spread with parameters used for DBS is warranted to formally exclude the effect on GPe. However, this effect is unlikely due to good clinical response of patients to the DBS and absence of the adverse effects.

Nevertheless, our results suggest that the GPI may be involved in both reactive and proactive inhibitory processes. To unravel the precise role of the GPI in these two forms of inhibitory control, future studies on animal models using intracranial recordings could be useful.

## Methods

Here we report how we determined our sample size, all data exclusions, all inclusion/exclusion criteria, all manipulations, and all measures in the study.

### Subjects

The study was approved by the ethics committee (CPP/AU-1360) and preregistered (https://clinicaltrials.gov/ct2/show/NCT03351218). Three groups of participants were recruited: 25 healthy controls (HC), 22 MD without GPI deep brain stimulation (MD group) and 12 MD patients with deep brain stimulation of GPI (MD-DBS group). Informed consent was obtained from all subjects or from the legal guardian.

Patients were recruited through the French Movement Disorders Clinical Network across France and all assessed at the Pitié-Salpêtrière Hospital (Paris). All patients had a proven *SGCE* mutation. HC were matched to MD patients in terms of gender, age, education level, and laterality assessed by Edinburgh Handiness Inventory^54^. All participants gave their written consent according to the Declaration of Helsinki. Prior to the data collection and after identification of all available patients for inclusion (MD is a rare disorder), we performed evaluation of the effect size to obtain significant results and found that our population allowed to find significant findings assuming moderate size effect.

Inclusion criteria for patients were a diagnosis of SGCE positive MD, no botulinum toxin injection for at least 3 months, stable pharmacological treatment in the month preceding inclusion. Inclusion criteria for HC were absence of chronic disorders and any kind of treatment, excluding birth control pills for women.

MD severity was assessed by the Burke–Fahn–Marsden scale^55^ for dystonia and by the Unified Myoclonus Rating Scale^56^ at rest and during action for myoclonus. For the MD-DBS group, pre- and post-surgery scores were collected. For all participants, additional information were collected for depression using the Beck Depression Inventory^57^, for the OCD and other Axis 1 disorders using the Mini International Neuropsychiatric Interview^58^ and for the impulsive behaviours using the Minnesota Impulse Disorders Interview^59^.

### Stop Signal Task

**Figure 3:**
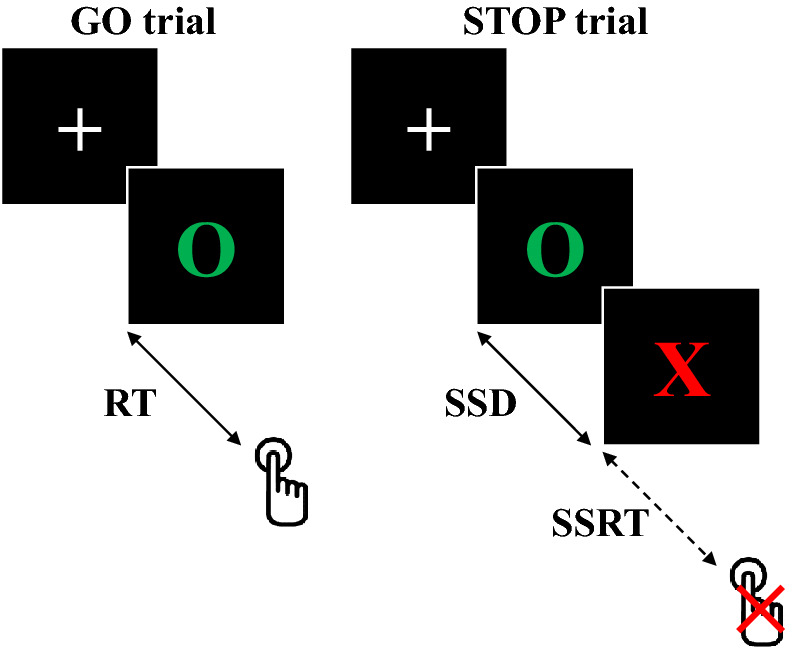
Schematic representation of GO and STOP trials during the Stop Signal Task. *RT* reaction time, *SSD* stop-signal delay, *SSRT* stop signal reaction time.

The Stop Signal Task^60^ was programmed using the E-Prime software^61^ and included 270 trials during a 30 min session. As illustrated in Fig. 3, the participants were installed in front of a laptop screen and were instructed to press to start the experiment. On a majority of trials (66.6%) the subjects were instructed to respond and press the keyboard as quickly as possible after the presentation of a GO-signal (green circle, GO-trials) appearing after a variable delay (from 10 to 170 ms). On 33.3% of trials, the participants were instructed to withhold their response when the GO-signal was followed by a STOP-signal (red cross, STOP-trials). A delay of 250 ms between the GO-signal and the STOP-signal presentations (stop-signal delay, SSD) were fixed for the first STOP trial. In the rest of the session, the stop-signal delays were adjusted by step of 25 ms as a function of the subject performance: using a staircase procedure^62^.

Performance in this paradigm has been modelled as a race between go and stop processes^60^. The presentation of GO and STOP signals activates the respective processes, which are considered to run independently. If the GO reaches the activation threshold before the STOP process, then the action will be executed. Conversely, if the STOP process exceeds the GO process then the execution of the action will stop. However, while the duration of the GO process is directly observable through the reaction time during the GO tests, the duration of the STOP process, called the stop signal reaction time, can only be estimated by observing the effects of varying the stop-signal delay: when the stop-signal delay is short, the action inhibition is easy to perform and probability of success is high. On the contrary, when the stop-signal delay is longer, the action is more difficult to inhibit and the probability of failure is high. Therefore, the stop signal reaction time could be interpreted as a measure of reactive inhibitory control.

Before calculating the stop signal reaction time, the inhibition function for each participant was analysed. This function represents the probability of response during a STOP trial (p(response|signal)) as a function of the stop-signal delay. All unexpected curves were excluded from the final analyses (i.e. if the inhibition curves did not reach a threshold of 25% of inhibitory failure, or if the proportion of inhibitory failure did not increase when the stop-signal delay increased). The stop signal reaction time were calculated using the integration method^60^ with replacement of GO omissions, which provides the least biased and most reliable stop signal reaction time estimates according to recent recommendations^23^. For the MD-DBS group, the SSRT was performed with DBS ON.

In addition, we considered if consecutive GO trials decreased reaction time and increased p(response|signal) and if consecutive STOP trials increased reaction time and decreased p(response|signal). We considered that these metrics represent proactive inhibitory control since they imply that participants change their behaviour at each trial according to the nature of the previous trials.

### Statistical analysis

All statistical analyses were performed with the statistical R software and the thresholds of significance have been set at *p* ≤ 0.05. Demographic data were analysed using χ^2^ and ANOVA. For the Stop Signal Task, we excluded outliers (mean stop signal reaction time ± 3 * standard deviation in each group) and used ANOVA and generalized linear mixed models (including subjects and trials as random effects) to study effects of groups (HC, MD and MD-DBS) and OCD comorbidity on the main measures: stop signal reaction time, reaction time and p(response|signal). Post-hoc analyses were performed using Tukey adjustment for multiple comparisons. To avoid Error II, we performed Bayesian analyses with 10,000 iterations for all statistically non-significant results.

Lastly, correlations corrected by permutations (n = 5,000) were performed among the significant effects and clinical data using Bonferroni testing for each observation based on Studentized residuals to identify possible outliers. If the outliers were identified, we performed a second correlation with permutations without outlier’s data to determine if correlation were still significant.

### Ethical statement

All experimental protocols were approved by the ethics committee (Comité de Protection des Personnes; CPP/AU-1360), preregistered (https://clinicaltrials.gov/ct2/show/NCT03351218) and conform with the Declaration of Helsinki.

### Methodological statement

All methods were carried out in accordance with relevant guidelines and regulations.

## Supplementary information

Supplementary Information.

## Data Availability

The conditions of our ethics approval do not permit public archiving of individual anonymised raw data. Readers seeking access to the data should contact the lead authors Drs. Worbe and Roze. Access will be granted to named individuals in accordance with ethical procedures governing the reuse of sensitive data. Specifically, requestors must obtain a specific authorization from the ethics committee.
