# Supplementary material for: Dissociation in reactive and proactive inhibitory control in Myoclonus dystonia
Source: Sci Rep. 2020 Aug 18;10:13933. doi: 10.1038/s41598-020-70926-x (PMC7434767; doi:10.1038/s41598-020-70926-x)
Supplement: Supplementary file 1 — Supplementary Information. [file 41598_2020_70926_MOESM1_ESM.docx]

**Dissociation in reactive and proactive inhibitory control in Myoclonus dystonia**

Cyril Atkinson-Clement PhD*^1^, Clement Tarrano MD*^1,2,3^, Camille-Albane Porte MSc^1^, Nicolas Wattiez MSc^4^, Cécile Delorme MD^1,2^, Eavan M. McGovern MD PhD^2,5^, Vanessa Brochard PhD^6^, Stéphane Thobois MD PhD^7^, Christine Tranchant MD PhD^8^, David Grabli MD PhD^1,2^, Bertrand Degos MD PhD^9^, Jean-Christophe Corvol MD PhD^2^, Jean-Michel Pedespan MD PhD^10^, Pierre Krystkoviak MD PhD^11^, Jean-Luc Houeto MD PhD^12^, Adrian Degardin MD PhD^13^, Luc Defebvre MD PhD^14^, Romain Valabregue PhD^1,15^, Charlotte Rosso MD PhD^1,16^, Emmanuelle Apartis MD PhD^1,17^, Marie Vidailhet MD^1,2^, Pierre Pouget PhD**^1^, Emmanuel Roze MD PhD**^1,2^, Yulia Worbe MD PhD**^1,17^

* / ** these authors contributed equally to this work

**1** Sorbonne University, 75005 Paris; Inserm U1127, CNRS UMR7225, UM75, ICM, F-75013, Paris, France. Movement Investigation and Therapeutics Team, Paris, France.

**2** Assistance Publique-Hôpitaux de Paris, Centre d'Investigation Clinique Neurosciences, Hôpital Pitié-Salpêtrière, Paris, France; Department of Neurology, Groupe Hospitalier Pitié-Salpêtrière, Paris, France.

**3** Department of Neurology, CHU Côte de Nacre, Université Caen Normandie, Caen, France.

**4** Sorbonne University, Inserm, UMRS1158 Neurophysiologie Respiratoire Expérimentale et Clinique, Paris, France.

**5** Department of Neurology, St Vincent's University Hospital Dublin, Dublin, Ireland.

**6** Centre d'Investigation Clinique 1422, INSERM/APHP, Paris, France.

**7** University of Lyon, Institut des Sciences Cognitives Marc Jeannerod, CNRS, UMR 5229, Bron, France; Hospices Civils de Lyon, Hôpital Neurologique Pierre Wertheimer, Service de Neurologie C, Bron, France.

**8** Service de Neurologie, Hôpitaux Universitaires de Strasbourg, Institut de Génétique et de Biologie Moléculaire et Cellulaire (IGBMC), INSERM-U964/CNRS-UMR7104/Université de Strasbourg, Fédération de Médecine Translationnelle de Strasbourg (FMTS), Université de Strasbourg, Strasbourg, France.

**9** Assistance Publique-Hôpitaux de Paris, Department of Neurology, Hôpital Avicennes, Bobigny, France.

**10** Unité de Neuropédiatrie, CHU Pellegrin, Bordeaux, France.

**11** Department of Neurology, Amiens University Medical Center, Amiens, France.

**12** Service de Neurologie, CIC-INSERM 1402, CHU de Poitiers, Poitiers, France.

**13** Department of Neurology, Centre Hospitalier de Tourcoing, Tourcoing, France.

**14** Université de Lille, CHU Lille, INSERM, U1171-Degenerative & Vascular Cognitive Disorders, Lille, France; Lille Centre of Excellence for Neurodegenerative Diseases (LiCEND), Lille, France.

**15** Centre de NeuroImagerie de Recherche (CENIR), Sorbonne Université, UMR S 975, CNRS UMR 7225, ICM, Paris, France.

**16** Assistance Publique-Hôpitaux de Paris, Urgences Cérébro-Vasculaires, Hôpital de la Pitié Salpêtrière, Paris, France.

**17** Department of Neurophysiology, Saint-Antoine Hospital, Assistance Publique-Hôpitaux de Paris, Paris, France.

**Corresponding author:**

Dr Yulia Worbe, MD, PhD

Sorbonne University, Department of Neurophysiology

Saint-Antoine Hospital, Paris, France

Email: yulia.worbe@aphp.fr

**Word count (excl. References):** 3424

**Abstract word count:** 197

**Ref:** 61

**Tables and Figures:** 5

**Running title:** Inhibitory control in Myoclonus Dystonia

**Key words:** Inhibition, Myoclonus dystonia, Deep brain stimulation, Internal globus pallidus

**Financial disclosure:** The authors report no conflict of interest.

**Funding sources:** The study received the financial support from the Dystonia Medical Research Foundation (Chicago, USA), program “Investissements d’Avenir” ANR-10-IAIHU-06, Fonds de Dotation Brou de Laurière, Association AMADYS, MERZ-Pharma and National Research Agency (ANR-18-CE37-0008-01).

**Supplementary Table.1: Demographics and clinical characteristics for (A) unoperated and (B) operated patients**

| **N** | **Gender** | **Age** | **Education years** | **Medication** |
| --- | --- | --- | --- | --- |
| **A. MD patients** | | | | |
| 1 | M | 32 | 14 | - |
| **2** | M | 40 | 17 | Zonisamide (250mg/d) |
| **3** | F | 37 | 13 | - |
| **4** | M | 20 | 13 | - |
| **5** | F | 23 | 12 | - |
| **6** | F | 19 | 14 | Trihexyphenidyl |
| **7** | M | 41 | 14 | - |
| **8** | M | 47 | 12 | - |
| **9** | M | 20 | 13 | - |
| **10** | F | 27 | 12 | - |
| **11** | F | 20 | 12 | - |
| **12** | M | 26 | 15 | Zonisamide (175mg/d) – Trihexyphenidyl (5mg/d) |
| **13** | M | 35 | 12 | Tetrabenazine (0.5cp/d) – Clonazepam (2mg/d) |
| **14** | F | 41 | 7 | Zonisamide (150mg/d) |
| **15** | M | 16 | 12 | - |
| **16** | M | 41 | 12 | Alprazolam – Clonazepam – Propranolol – Paroxetine |
| **17** | M | 21 | 15 | - |
| **18** | F | 19 | 13 | Clonazepam (15drops/d) |
| **19** | M | 23 | 15 | - |
| **20** | F | 60 | 14 | - |
| **21** | M | 23 | 15 | Trihexyphenidyl |
| **B. MD-DBS patients** | | | | |
| **22** | F | 35 | 14 | - |
| **23** | M | 52 | 12 | Clonazepam (2mg/d) |
| **24** | M | 28 | 8 | - |
| **25** | F | 24 | 12 | - |
| **26** | M | 54 | 14 | Clonazepam (2mg/d) |
| **27** | F | 23 | 12 | Sertraline (50mg/d) – Zonisamide (100mg/d) |
| **28** | F | 38 | 15 | - |
| **29** | M | 29 | 12 | - |
| **30** | M | 32 | 12 | - |
| **31** | F | 31 | 12 | - |
| **32** | F | 40 | 12 | - |
| **33** | M | 22 | 12 | - |

F: Female; M: Male; MD: Myoclonus-dystonia without DBS; MD-DBS: Myoclonus-dystonia with DBS.

**Supplementary Table.2: Deep brain stimulation parameters for operated patients.**

|  | **Left** | | | | **Right** | | | |
| --- | --- | --- | --- | --- | --- | --- | --- | --- |
| **N** | **V** | **Hz** | **µs** | **Contacts** | **V** | **Hz** | **µs** | **Contacts** |
| **22** | 3.6 | 130 | 60 | - | 3.6 | 130 | 60 | - |
| **23** | 2.4 | 130 | 60 | 4 | 3.1 | 130 | 60 | 1 |
| **24** | 3 | 160 | 90 | 1 | 3 | 160 | 90 | 1 |
| **25** | 2.6 | 130 | 60 | 1 | 3.3 | 130 | 60 | 1 |
| **26** | 2.4 | 130 | 60 | 5 | 2.5 | 130 | 60 | 1 |
| **27** | - | - | - | - | - | - | - | - |
| **28** | 3 | 130 | 90 | 10 | 2.9 | 130 | 90 | 3 |
| **29** | - | - | - | - | - | - | - | - |
| **30** | 2.5 | 125 | 60 | 10 | 2.5 | 125 | 60 | 3 |
| **31** | 3.5 | 130 | 60 | 9 | 3.2 | 130 | 60 | 1 |
| **32** | 2.7 | 130 | 80 | 1 | 3.6 | 130 | 60 | 2 |
| **33** | 2.4 | 130 | 60 | 10 | 2.4 | 130 | 60 | 2 |

-: Missing data; Contacts: locations of the deep brain stimulation current delivery on the four possible contacts along the electrode (generator case positive, electrode contact negative); Hz: Frequency in hertz; μs: Pulse width in microseconds; SD, standard deviation; V: Voltage in volts.

**Supplementary Figure.1: Power calculation before the study for an estimated sample of 36 patients and 36 controls.**


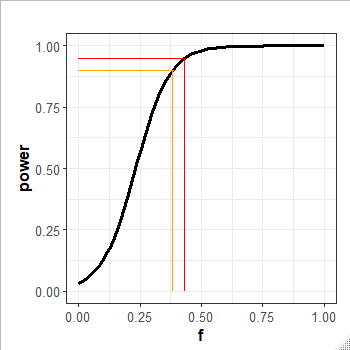


Orange line represents the f effect size needed for a power of 90% (f=0.39). Red line represents the f effect size needed for a power of 95% (f=0.44).
